# Nanoparticles and female reproductive system: how do nanoparticles affect oogenesis and embryonic development

**DOI:** 10.18632/oncotarget.19087

**Published:** 2017-07-07

**Authors:** Cong-Cong Hou, Jun-Quan Zhu

**Affiliations:** ^1^ College of Marine Sciences, Ningbo University, Ningbo, Zhejiang, China

**Keywords:** nanoparticles, toxicology, oogenesis, embryonic development, inflammation

## Abstract

Along with the increasing application of nanoparticles (NPs) in many walks of life, environmental exposure to NPs has raised considerable health concerns. When NPs enter a pregnant woman’s body through inhalation, venous injection, ingestion or skin permeation, maternal toxic stress reactions such as reactive oxygen species (ROS), inflammation, apoptosis and endocrine dyscrasia are induced in different organs, particularly in the reproductive organs. Recent studies have shown that NPs disturb the developing oocyte by invading the protective barrier of theca cells, granulosa cell layers and zona pellucida. NPs disrupt sex hormone levels through the hypothalamic–pituitary-gonadal axis or by direct stimulation of secretory cells, such as granule cells, follicle cells, thecal cells and the corpus luteum. Some NPs can cross the placenta into the fetus by passive diffusion or endocytosis, which can trigger fetal inflammation, apoptosis, genotoxicity, cytotoxicity, low weight, reproductive deficiency, nervous damage, and immunodeficiency, among others. The toxicity of these NPs depend on their size, dosage, shape, charge, material and surface-coating. We summarize new findings on the toxic effect of various NPs on the ovary and on oogenesis and embryonic development. Meanwhile, we highlight the problems that need to be studied in the future. This manuscript will also provide valuable guidelines for protecting the female reproductive system from the toxicity of NPs and provide a certain reference value for NP application in the area of ovarian diseases.

## INTRODUCTION

Nanoparticles (NPs) are characterized as particles with sizes between 1 to 100 nm. NPs exist in the natural environment due to biological, physical and chemical processes [1]. Moreover, due to their properties, including small particle size and a relatively high surface area, numerous applications of NPs are likely to further increase human exposure to NPs through inhalation, ingestion and dermal absorption, among other mechanisms [2, 3]. It cannot be ignored that the exposure to nanomaterials is potentially hazardous to the human body and environment. The negative impact of NPs on human health depends on individual factors, such as genetics and existing diseases as well as on the properties of NPs such as shape, size, structure, and inorganic and organic coatings [4, 5]. In recent years, many studies have reported toxic effects of NPs, mainly consisting of metal nanomaterials, nanometer oxides, carbon NPs and quantum dots [6]. Recent studies have shown that NPs can penetrate cells and subsequently disrupt their biological structures and normal functions via generation of ROS or by increasing intracellular oxidative stress [3]. The toxic effects induced by NPs include tissue inflammation and the imbalance of the cellular redox state, resulting in apoptosis or cell death [7-9]. Moreover, some NPs can penetrate the blood-testis barrier, placental barrier and blood-brain barrier and accumulate in different cells [10-12]. Reproductive toxicity caused by NPs is gradually rising in importance.

The studies on the toxicity of NPs in the female reproductive system mainly include effects on reproductive ability, teratogenic effects during embryonic development and impact on the offspring during the perinatal period [12, 13]. Recently, studies have shown that inhaled, ingested or dermally absorbed NPs are able to translocate through the circulatory system and even accumulate in different reproductive organs and in the fetus [14, 15]. Moreover, the reproductive toxicity of NPs in different germ cell lines *in vitro* and animal models *in vivo* are being increasingly reported. For example, *in vitro* studies showed that some NPs could be swallowed by granulosa cells, resulting in changes in the secretion of hormones and dysplasia of the ovum [16]. There are well-documented studies showing that NPs can enter both thecal cells and granule cells and affect their normal function, particularly relating to their key role in hormone secretion [16]. Before ovulation, androgens and androstenedione secreted by thecal cells diffuse into granule cells and are transformed into steroid hormones. During this process, NPs can directly affect the secretion of sex hormones by destroying these secretory cells in the ovaries [17, 18]. *In vivo* studies have shown that long-term (90 consecutive days) exposure to titanium dioxide NPs (TiO_2_ NPs) in female mice results in an imbalance of sex hormones and mineral element distribution, leading to a reduction in pregnancy rate and oxidative stress and disruption of ovarian gene expression [19, 20]. Moreover, *in vivo* experiments in rats showed that silver NPs of 34.9 ± 14.8 nm in size could get transferred from the mother to offspring through the placenta and breast milk [20].

Oogenesis, or ovum production, is the process of development from oogonia to a mature egg occurring in ovarian follicles [21]. Follicular atresia is a natural physiological process for follicle development, follicle maturation and ovulation in mammals. The regulated hormones of follicle atresia include estrogen, gonadotropin-releasing hormone (GnRH), gonadotropic hormone, androgen and growth hormone, among others [22, 23]. A typical follicle consists of the following layers (from the outside to inside): a thecal cell layer, basement membrane, granulosa cell layer, zona pellucida and oocyte. Recent reports showed that NPs could be endocytosed by thecal cells and granulosa cells, leading to abnormal hormone secretion and termination of oocyte development *in vivo* [17]. *In vitro* experiments also suggested that the engulfment of 10 nm nanogold particles by granulosa cells could result in steroidogenesis imbalance and ovum dysplasia [16]. In other words, NPs of a particular size can accumulate in secretory cells and directly affect hormone secretion in the ovaries. However, there is no evidence showing that NPs can actually penetrate oocytes and accumulate inside them as of yet. The reason for this may be attributed to the multiple layers of follicles and the special structure of zona pellucida [24].

NPs may cross or circumvent the blood-brain barrier (BBB) and accumulate in the central nervous system (CNS) [25]. One of the potential harmful impacts from such stimulation of the nervous system by NPs is the disruption of hormone secretion. The neurohormones such as GnRH, follicle stimulating hormone (FSH) and luteinizing hormone (LH) secreted by the hypothalamus and pituitary play crucial roles in positive and negative feedback regulation during oogenesis. NPs may indirectly affect oogenesis and ovarian health by disturbing the balance of these sex hormones [25].

As commercial uses for nanotechnology and nanomaterials continue to increase across the world, the risk for unintentional exposure or purposeful application of NPs in the workplace increases, and more and more NPs can have potential generational impacts or disrupt embryonic development [27]. For instance, exposure to gold NPs (Au NPs) was reported to alter the expression of 19 genes in human fetal lung fibroblasts [26]. In this review, we mainly focus on the toxic effect of NPs on the female reproductive system and embryonic development. This review will provide guidelines for taking care of vulnerable populations such as women working in manufacturing industries. Additionally, it will provide appropriate references for reducing the risk of nanomaterial application and support reasonable industry development.

## TOXICITY OF NPS IN OOGENESIS

The toxicity of NPs vary with their size, charge, surface-coating, material, concentration, exposure time and tolerance in different cell types or animal models. Most reports on the toxicity of NPs in the female reproductive system mainly include metal, metal oxide, carbon particles and CdSe-core quantum dots (QDs) (Tables 1-4). *In vivo* and *in vitro* studies showed that certain sizes of NPs could penetrate different female germ cells and accumulate inside them, which initiate different cell responses such as oxidative stress [28], DNA damage [28], apoptosis [29], inflammation [30] and inhibition of signal transduction [21].

**Table 1:**
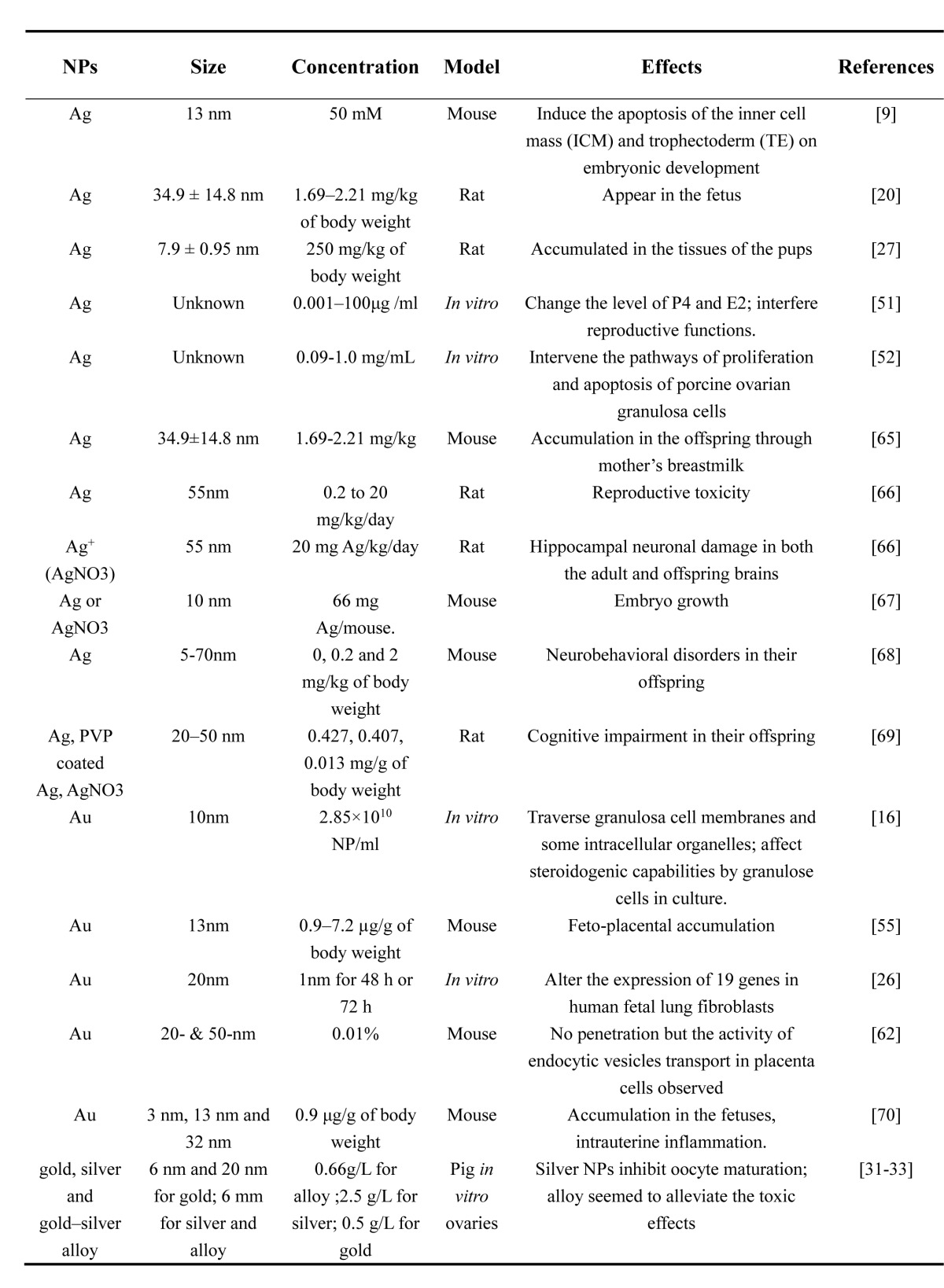
Toxicity of Ag and Au NPs on ovary, oogenesis and embryonic development

**Table 2:**
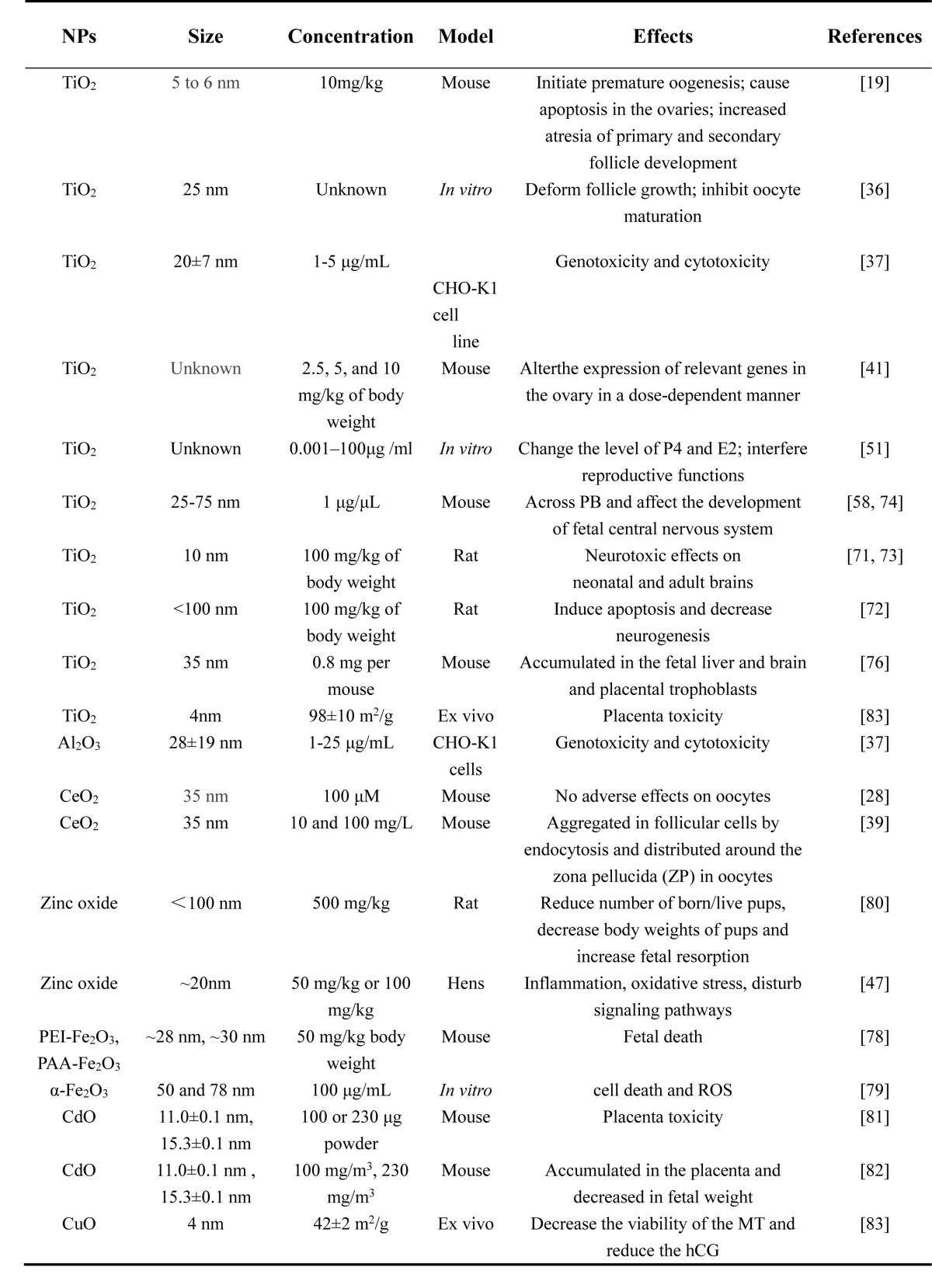
Toxicity of metallic oxide NPs on ovary, oogenesis and embryonic development

**Table 3:**
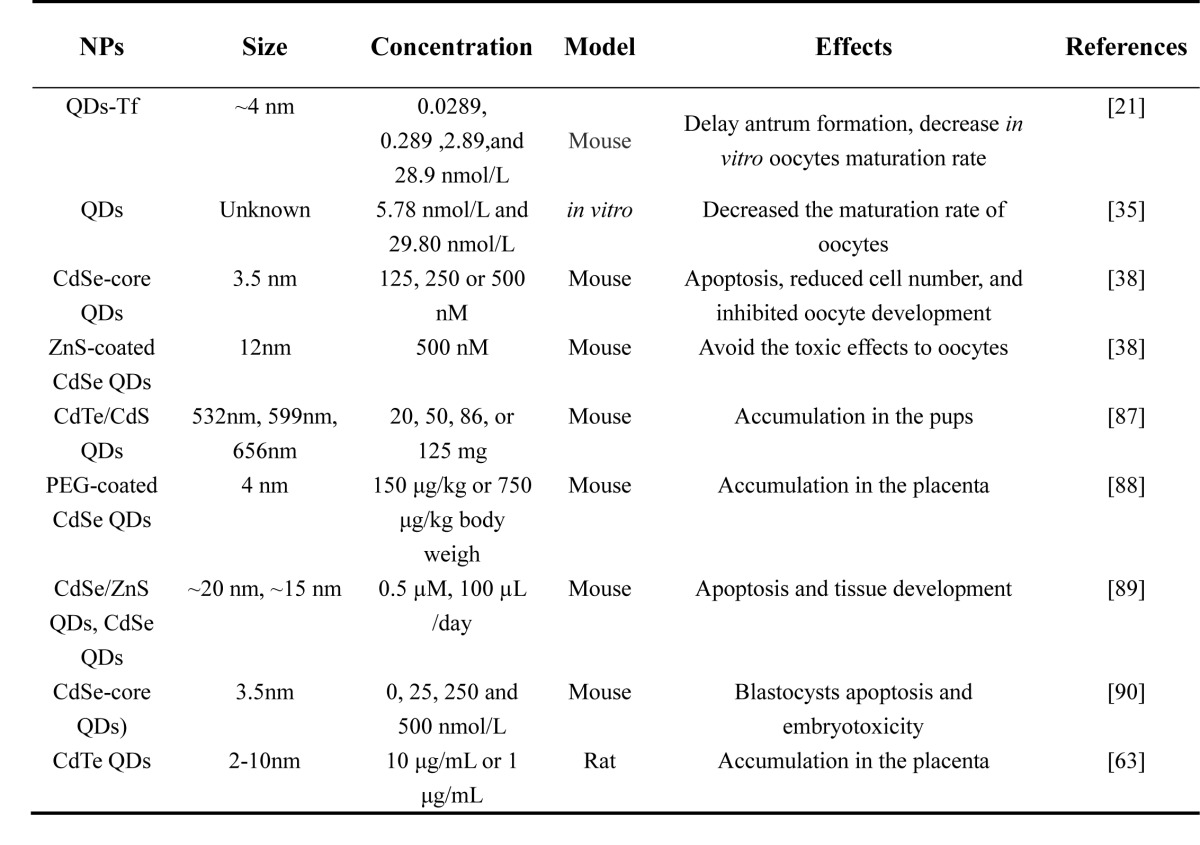
Toxicity of QDs NPs on ovary, oogenesis and embryonic development

**Table 4:**
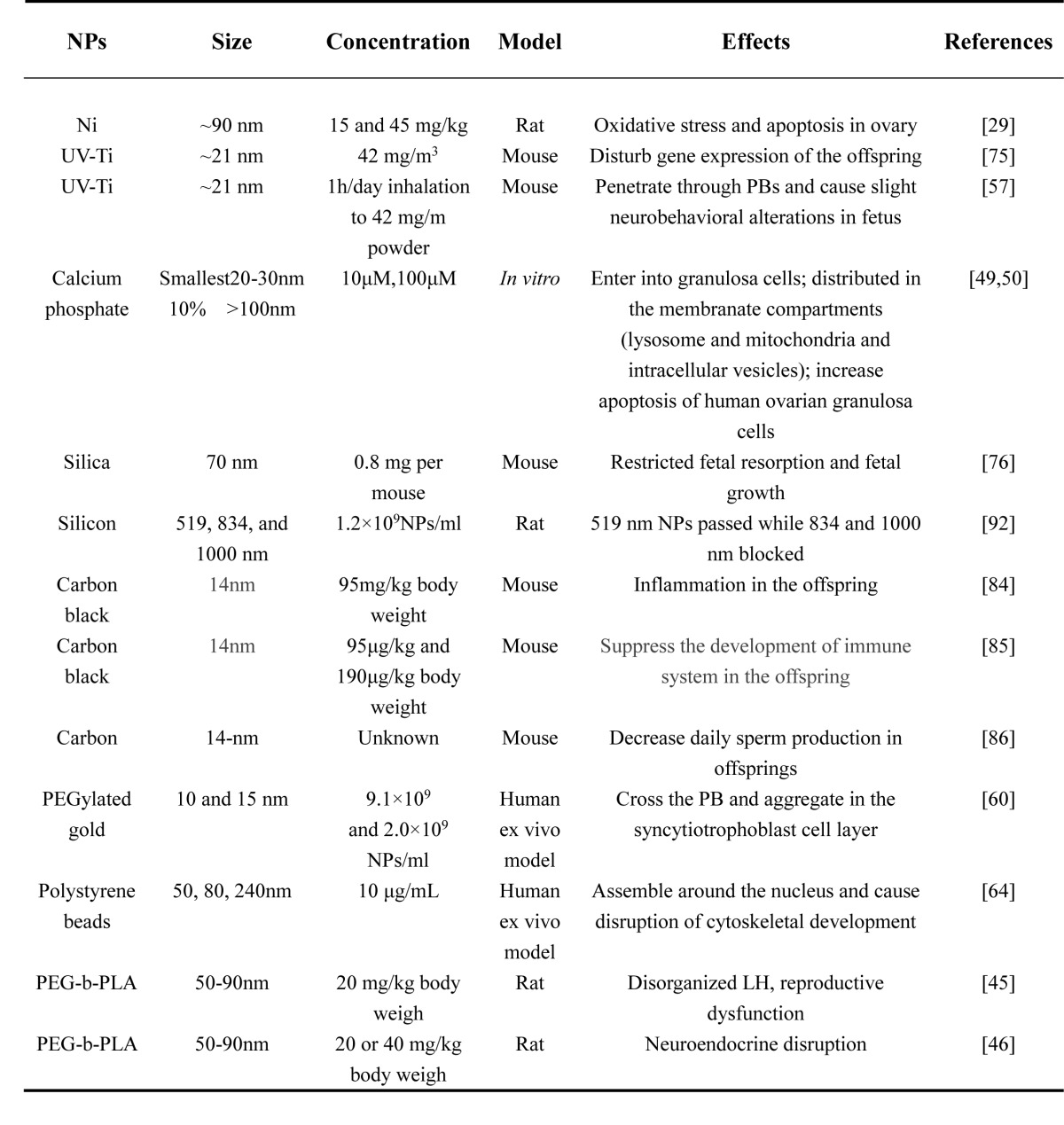
Toxicity of other NPs on ovary, oogenesis and embryonic development

### *In vitro* studies

It is known that growth and maturation of oocytes are extremely sensitive to micro-environmental changes, particularly to extracellular chemical compounds. Recent studies have demonstrated the novel phenomenon that mammalian oocytes display different toxic responses to gold, silver and gold-silver alloy NPs [31]. The results showed that both silver and gold NPs mostly accumulate in the cumulus cell layers and oocytes [31]. Additionally, studies provided evidence that the toxicity to oocytes increased with the silver molar fraction [31-33]. An *in vitro* model of zebrafish ovarian follicle cells surrounding an oocyte, thecal cells and granulosa cells exhibited apoptosis with phenotypes of irregular cell morphology, disorganized cytoplasm and fragmented and condensed nuclei after exposure to Ag NPs (30 mg/mL) and AgNO_3_ (10 mg/mL) [34]. *In vitro* experiments also showed that quantum dot-transferrin (QD-Tf) bioconjugates were mostly taken up by the cumulus cells, but no QDs entered the oocytes. With an increasing concentration of QD-Tf bioconjugates, antral formation was delayed and the *in vitro* maturation rate of oocytes displayed an obvious decrease (from 62% to 21.8%) [21]. The authors deduced that both QDs and QD-Tf bioconjugates exhibited reproductive toxicity. They concluded that the mechanism of interference with oogenesis was mediated by the disturbance of antral formation in oocytes, dysfunction of cumulus cells or disruption of signal transduction between germ cells and somatic cells [21, 35]. Fortunately, the analysis of chromosomal abnormalities demonstrated that nuclear maturation in the oocytes was unaffected by the QD-Tf bioconjugates [21]. TiO_2_ NPs have also been reported to cause ovarian developmental disorders in mammals. In one study, a rat preantral follicle exposed to 25 nm TiO_2_ at 25 μg/ml exhibited deformed follicular growth and inhibited oocyte maturation [36]. Dose-related genotoxicity and cytotoxicity of TiO_2_ and Al_2_O_3_ NPs have been noted in Chinese hamster ovarian cells (CHO-K1). In that study, the adverse effect of TiO_2_ was observed at lower concentrations and was noted to be stronger than that due to exposure of Al_2_O_3_ NPs [37]. A previous study showed that QD formulations were non-toxic to cancer cells, while more recent studies reveal that exposure to QDs can decrease the maturation rate of oocytes [35]. To detect the toxicity of CdSe-core QDs (3.5 nm) in oocytes, the oocytes collected from 21-day-old mice were treated with CdSe-core QDs (CdSe; 125, 250 or 500 nM) or ZnS-coated CdSe QDs (ZnS/CdSe; 500 nM). Some oocytes treated with CdSe-core QDs underwent apoptosis, while others exhibited reduced cell number, and oocyte development was inhibited during *in vitro* maturation (IVM). It is worth noting that the surface coating of ZnS could avoid these toxic effects of CdSe-core QDs on oocyte maturation and embryonic development [38]. This study indicated that the surface coating of NPs would alter their toxicity.

It was observed that CeO2 NPs had accumulated in follicular cells by endocytosis and were distributed around the zona pellucida (ZP) in oocytes [28]. At high concentrations of CeO_2_ NPs, follicular cell endocytosis and zona pellucida trapping were unable to protect mature oocytes from oxidative stress and DNA damage [28]. During *in vitro* fertilization (IVF), mouse oocytes cultured with medium containing CeO_2_ NPs at very low concentration (0.01 mg/l) showed a significantly lower fertilization rate compared with that of the control group [39]. The reasons of low fertilization rate may be genotoxicity and oxidative stress in gametes due to CeO_2_ [39]. Comet assay also showed that oocytes cultured with CeO_2_ showed significant DNA damage at the same low concentration [39]. At a high concentration (100 mg/l), CeO_2_ NPs penetrated the cumulus cells surrounding oocytes by endocytosis and were accumulated along the zona pellucida of oocytes [39]. As CeO_2_ engineered NPs (ENPs) have excellent biomedical properties for potential use in the treatment of endometriosis and protection of endometriosis-related adverse effects on oocytes [40], the results from the two abovementioned *in vitro* studies may provide evidence for the need for minimizing toxicity in the medical applications of CeO_2_ NPs.

### *In vivo* studies

During the process of follicular development, theca cells, granulosa cell layers and the zona pellucida serve as protective barriers to prevent exogenous substances from entering the developing oocyte. Therefore, the layers of theca cells and granulosa cells are injured before the oocyte. Studies on female mice showed an accumulation of TiO_2_ NPs in the ovaries and the consequent initiation of premature oogenesis. Such abnormal processes can potentially result in malformed ova and in the induction of reproductive system dysfunction. To be specific, accumulation of NPs can cause apoptosis in the ovaries, a process potentially mediated by the activation of BCL2 modifying factor (BMF) and the mitochondria-mediated apoptotic pathway [19]. As mentioned previously, most of the ovarian follicles undergo atresia during development, which is a process of hormonally controlled apoptosis regulated by various factors [22, 23]. With long-term exposure to TiO_2_ NPs, the expression levels of 288 genes involved in hormone and cytokine pathways were found to have been changed in the mouse ovary [41]. The authors suggested that the ovarian inflammation and follicular atresia may have been due to nano-TiO_2_ in a dose-dependent manner, altering the expression of relevant genes in the ovary [41]. In an *in vivo* study, female rats exposed to Ni NPs (∼90 nm) at doses of 15 and 45 mg/kg exhibited reproductive toxicity, such as mitochondrial swelling, disappearance of mitochondrial cristae, and enlargement of the endoplasmic reticulum in ovaries [29]. Due to the signi?cantly decreased activity of superoxide dismutase (SOD) and catalase (CAT) and increased ROS, malondialdehyde (MDA) and NO, as well as the increased expression of pro-apoptotic proteins such as Fas, AIF, Cyt c, Bax and Bid, the possible damage mechanism may have been due to oxidative stress and apoptosis induced by the Ni NPs [29].

**Figure 1:**
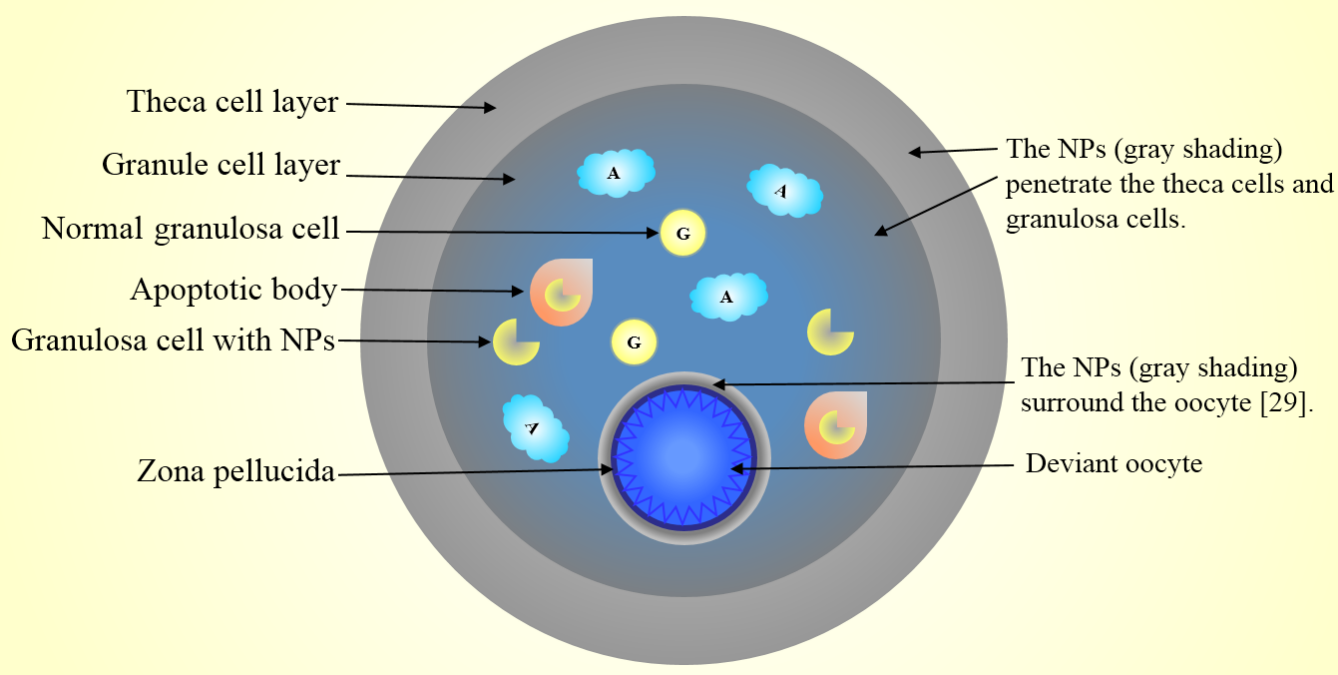
Nanoparticles penetrate developing follicles and interfere with the maturation of the oocytes NPs accumulate in the cytoplasm and nuclei of theca cells and granule cells. The accumulation of NPs results in ovarian cell apoptosis and accelerates the formation of antrum. Major NP accumulation occurs in the cumulus cell layer surrounding the oocyte, but no NPs enter the oocytes due to trapping by the zona pellucida.

In summary, NP accumulation occurs in the cumulus cell layer surrounding the oocyte, but no NPs enter the oocytes due to trapping by the zona pellucida. NPs mainly accumulated in the cytoplasm and nuclei of theca cells and granule cells which resulted in ovarian cell apoptosis and antrum formation (Figure 1).

## THE EFFECTS OF NPS ON STEROIDOGENESIS

In a mature female, balanced hormone levels are crucial for maintaining healthy ovaries and supporting pregnancy. Reproductive function, fertility and the process of oogenesis are ultimately regulated by the hypothalamic-pituitary-gonadal axis [42]. Gonadotropin-releasing hormone (GnRH), secreted by the hypothalamus, can regulate the secretion of FSH and LH by the pituitary gland. FSH and LH are then transferred to the ovary via blood circulation and subsequently regulate the secretion of estrogen and progesterone in the ovaries to maintain normal menstruation and reproduction [42]. Such hormonal regulation is sensitive to allogenic materials such as NPs. NPs have been found to be able to disrupt sex hormone levels through the hypothalamic-pituitary-gonadal axis or by direct stimulation of secretory cells, such as granule cells, follicle cells and thecal cells and the corpus luteum [43].

### Neuroendocrine dysfunctions triggered by NPs

The function of the hypothalamic-pituitary-ovarian axis (HPOA) is to adjust and control the secretion of neurohormones, such as GnRH, LH and FSH, which regulate the reproductive system in females. Inhaled NPs have been shown to cross or circumvent the BBB via systemic circulation and subsequently accumulate in the CNS [25]. The exposure of NPs in the female may affect the functions of the HPOA, and thereby increase the risk of neurohormone imbalance. In an *in vivo* study, female rats exposed to Ni NPs (∼90 nm) at doses of 15 and 45 mg/kg displayed sex hormonal imbalance (increasing FSH and LH and decreasing estradiol) and ovarian damage (increasing apoptotic cells, inflammatory cell infiltration, lymphocytosis and vascular dilation and congestion) [44]. Female Wistar rats from postnatal day 4 to day 7 were daily injected intraperitoneally with poly(ethylene glycol)-block-poly(lactic acid) (PEG-b-PLA) NPs (20 mg/kg) [45]. The results indicated that the neonatal rats exposed to PEG-b-PLA NPs appeared to have impaired reproductive system development with disordered LH release by the pituitary, leading to reproductive dysfunctions during adulthood [45]. Female Wistar rats from postnatal days 4 to 176 were intraperitoneally injected with PEG-b-PLA at a dose of 20 or 40 mg/kg [46]. The authors observed that the hypothalamic gonadotropin-releasing hormone-stimulated luteinizing hormone secretion was altered and serum progesterone levels were increased and thus speculated that the neuroendocrine disruption was due to the PEG-b-PLA NPs [46]. Ingestion of ZnO (50 mg/kg or 100 mg/kg) could alter gene and protein expression of neuronal factors in the ovary, thereby regulating the population of neuroendocrine cells in the ovary [47]. One possible reason is that the accumulation of NPs in neurosecretory cells cause inflammation or oxidative stress, or disturb signaling pathways to perturb positive and negative feedback regulations in the HPOA, as well as the normal functions of neuroendocrine cells in females [44, 47]. Hormonal imbalance in adult females results in menstrual dysfunction, infertility, or abortion. However, the mechanisms by which NPs alter the functions of HPOA ultimately resulting in female infertility have not been investigated thoroughly. Meanwhile, the amount of researches about NPs having negative effects on HPOA is relatively small. It is certain that NPs with size of 36 nm were significantly accumulated in cerebrum and cerebellum translocation via the olfactory nerve and increased with the exposure time [25]. The sizes of NPs less than 90nm could disturb the balance of GnRH, FSH and LH, such as Ni, PEG-b-PLA [29, 45-46].

### Steroid hormone imbalance triggered by toxicity of NPs

In the ovaries, estrogen and progesterone are the two main types of steroid hormones. A small amount of androgen is also secreted. Superabundant steroidogenesis in the ovaries can lead to abnormal ovarian pathology, such as polycystic ovarian syndrome [48]. Conversely, reduced steroidogenesis in the ovary can also lead to abnormal follicle growth, even anovulation, resulting in infertility [17].

Exposure to TiO_2_ NPs can significantly alter serum levels of sex hormones, including progesterone (P4), LH, T, FSH and estradiol (E2), resulting in increased atresia of the primary and secondary follicle development and reduced fertility [19]. Ovarian granulosa cells are also involved in steroidogenesis and are important for maintaining ovarian function. *In vitro* studies showed that gold particles could traverse granulosa cell membranes and certain intracellular organelles, such as lipid droplets and mitochondria [16]. The gold particles could negatively affect the steroidogenic capabilities of granulose cells in culture. After 24 h, the levels of estradiol-17 beta, secreted by granulosa cells, also exhibited significant fluctuations compared to basal control levels [16]. This factor may be regulated by steroidogenic enzyme side-chain cleavage (SCC), located on the inner membrane of mitochondria [18]. This study is consistent with a later study that demonstrated that QD-Tf bioconjugates could also penetrate the first protective layer of the follicle and accumulate in the cytoplasm of theca cells and granulosa cells. This might have also altered the activity of the mitochondrial inner membrane enzyme SCC to disturb steroid biosynthetic pathways [21]. Calcium phosphate hydroxyapatite (HA) NPs are now one of the most widely used medicinal materials in the bone-repair field [49]. Due to their small size and unique properties, HA NPs can enter granulosa cells in culture and are distributed throughout the membrane compartments, including the lysosome, mitochondria and intracellular vesicles. Accumulation of calcium phosphate NPs were shown to perturb the cell cycle of cultured human ovarian granulosa cells resulting in increasing cell apoptosis [50]. To detect the effects of different NPs on steroid hormone secretion in porcine ovarian granulosa cells *in vitro*, metal NPs including TiO2 and Ag NPs (0.001-100 µg/ml) were used as inducers [51]. The results indicated that the two kinds of NPs induced changes in steroid hormone secretion (P4 and E2) by ovarian granulosa cells, resulting in the interference of reproductive function [51]. *In vitro*, exposure to Ag NPs at doses of 0.09-1.0 mg/mL interfered with pathways involved in proliferation and apoptosis of porcine ovarian granulosa cells, including the secretion of growth factor IGF-I and proliferation marker cyclin B1 and apoptosis marker caspase-3 [52].

P4 is a type of steroid hormone involved in the menstrual cycle, pregnancy and embryogenesis [53]. P4 is produced by the ovary, adrenal gland, placenta and corpus luteum. The corpus luteum is the major source of P4 during pregnancy [54]. The corpus luteum is important for steroidogenesis, which is required to maintain ovarian function and support gestation in pregnant women. However, it has been shown that the inhalation of nanoparticle-rich diesel exhaust by pregnant rats could suppress the function of the corpus luteum, resulting in a significantly decreased serum concentration of maternal P4 and subsequent increase in LH, corticosterone and estradiol-17β. This type of sex hormone imbalance was shown to increase the risk of spontaneous abortion in pregnant rats [15].

As we know, polycystic ovary syndrome (PCOS) is a heterogeneous disease with a variety of clinical manifestations. The aetiological agent is related to inheritance, environmental nutrition, unhealthy lifestyle, among others. One of its possible mechanisms is caused by endocrine disorders. GnRH pulse frequency, LH/FSH ratio are the possible reasons result to PCOS. Although NPs can lead to secretion disorders of GnRH, LH and FSH, there is no direct evidence demonstrating the association between NPs toxicity and this disease. In other words, whether women exposed to dust with NPs is more likely to have PCOS also need more researches.

**Figure 2:**
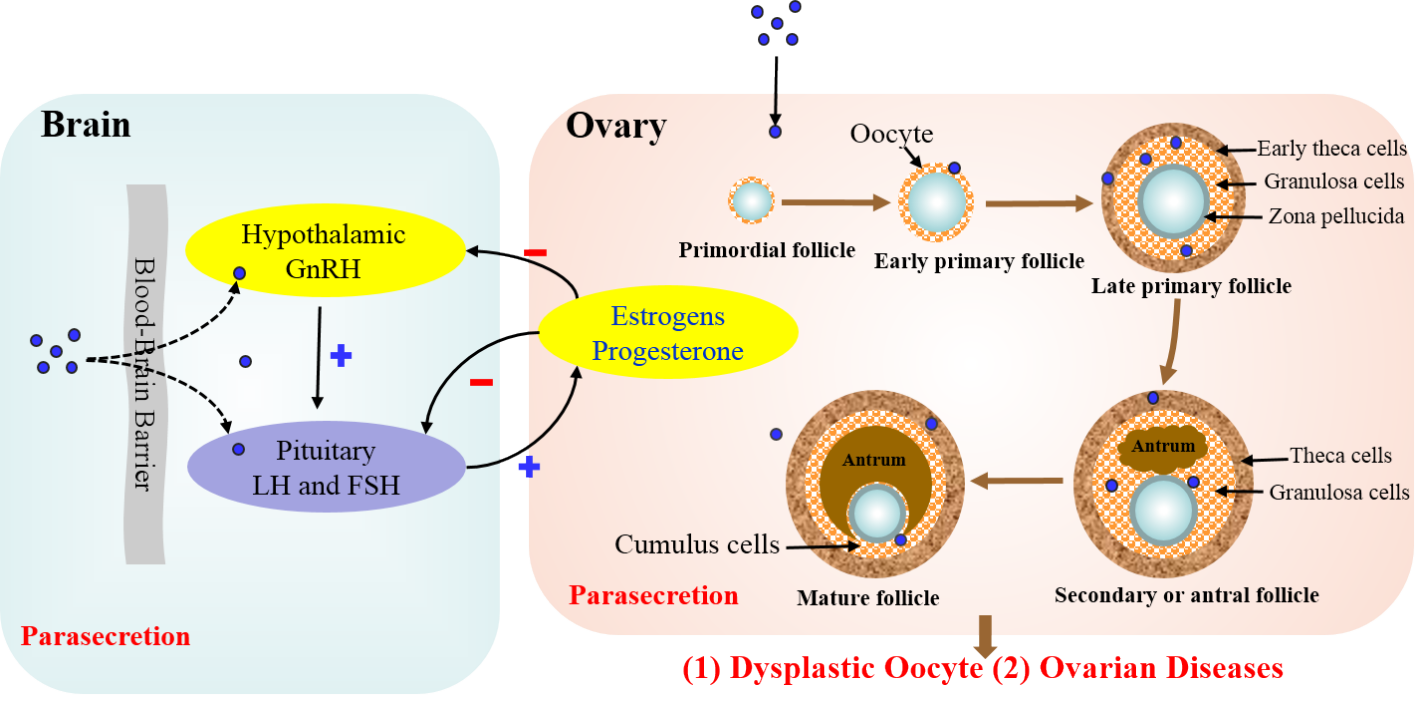
Effects of NPs on hormone secretion on the ovary and hypothalamic-pituitary-gonadal axis NPs may affect hormone secretion in two ways: 1. NPs pass through the blood-brain barrier into the hypothalamus and secretory cells of the pituitary altering the secretion of GnRH, LH and FSH, thus undermining the normal positive and negative feedback of the hypothalamic-pituitary-gonadal axis and affecting the normal secretion of ovarian estrogen and progesterone. 2. NPs enter the ovaries through circulation and accumulate in theca cells and granulosa cells, which affects steroidogenesis. This parasecretion eventually leads to dysplastic oocyte and ovarian diseases.

In summary, the effects of NPs on hormone secretion on the ovary and hypothalamic-pituitary-gonadal axis could be in two ways: 1. NPs pass through the blood-brain barrier into the hypothalamus and secretory cells of the pituitary altering the secretion of GnRH, LH and FSH, thus undermining the normal positive and negative feedback of the hypothalamic-pituitary-gonadal axis and affecting the normal secretion of ovarian estrogen and progesterone. 2. NPs enter the ovaries through circulation and accumulate in theca cells and granulosa cells, which affects steroidogenesis. This parasecretion eventually leads to dysplastic oocyte and ovarian diseases (Figure 2).

## TOXICITY OF NPS TO THE PLACENTAL BARRIER AND EMBRYONIC DEVELOPMENT

In murine fetuses, the concentration of NPs was markedly decreased in a manner concurrent to the maturation of placental barrier (PB). This suggested that the PB prevents the penetration of NPs [55]. Nonetheless, the concern for the potential effect of NPs on fetal health remains due to the sensitivity of the fetus to many toxins. Several studies found that NPs could traverse the rodent PB and result in varying levels of fetal harm (Tables 1-4).

The PB consists of three different layers of cells, namely, the syncytiotrophoblast, cytotrophoblast and fetal capillary endothelium. The PB is an important system in the exchange of materials between the mother and fetus. Additionally, the placental barrier acts as a natural barrier, as it prevents harmful substances from invading the fetus [56]. Generally, the placental barrier is formed at approximately GD10-12 in mice and at approximately 4 months of pregnancy in humans [27]. However, some NPs may cross the placental barrier and harm the fetus. In several studies, NPs were observed to penetrate the PB, while in other studies, the NPs were seen to be trapped in the PBs. For NPs that could reach the fetal tissues, some were suspected to harm fetal development and alter gene expression in the fetus, while some had no influence on the fetus [57, 58]. These diverse results are mainly due to the different size, coatings, and types of NPs [59]. Additionally, in order to study the effects of NPs on embryonic development as well as their application in fetal treatment, *in vivo* and *ex vivo* models of the human PB have been recommended [60, 61]. Nevertheless, the molecular transport mechanisms of NP penetration across the placental barrier are still poorly understood. There could be many potential pathways such as diffusion, passive transport, active transport, or the hypothetical ‘transtrophoblastic channel’ [62-64].

### Metal nanomaterials

Pregnant (20 days old) and lactating (14-16 days old) female Wistar rats were intragastrically administered with [^110m^Ag]-labeled Ag NPs (34.9 ± 14.8 nm) at doses of 1.69-2.21 mg/kg. After labeling for the following 24 and 48 h, a low-background semiconductor gamma-ray spectrometer was used to evaluate the accumulation of Ag NPs in the offspring and nursing pups consuming their mother’s breastmilk [65]. The results showed that the average level of accumulation of NPs in the fetuses was 0.085-0.147% of the administered dose, which exceeded the accumulation of NPs in the brains of females by at least 10-100 times and was comparable to the penetration of the NPs into tissues such as the liver, blood and muscles [65]. During the 48 h period of lactation, the total accumulation of [^110m^Ag]-labeled Ag NPs in the milk was at least 1.94 ± 0.29% of the administered dose, and the amount absorbed by the pups from the lactating mother was at least 25%, which indicated that Ag NPs could penetrated the PB and get transferred from the mother to offspring through breastmilk [65].

To evaluate the potential developmental toxicity of silver in both Ag NPs and Ag^+^ ionic forms, pregnant rats (7-20 days old) were orally administered both ionic Ag^+^ (AgNO_3_, 20 mg Ag/kg/day) and Ag NPs (55 nm, from 0.2 to 20 mg/kg/day). The results showed that oral administration of AgNO_3_ led to an increase in glutathione peroxidase (GPx) activity in both the adult and offspring brains resulting in hippocampal neuronal damage. Moreover, the Ag level in both maternal and offspring blood in the AgNO_3_-treated group was significantly higher than that of the Ag NP-treated group, which indicated that 1. both Ag+ ions and Ag NPs could translocate across the PB and 2. The reproductive toxicity of AgNO_3_ was higher than that of Ag NPs [66]. In a similar study, pregnant mice (7-, 8- and 9-days old) were intravenously injected with 10 nm Ag NPs or AgNO_3_ at a dose of 66 mg Ag/mouse. The results showed that the accumulation of NPs in the different tissues of mice treated with 10 nm Ag NPs was more on gestation day (GD) 10 than that on GD16, and the Ag concentration in the visceral yolk sac (VYS) of AgNO_3_-treated group was 2-fold that in the Ag NP-treated group. The only significant reproductive parameter was that approximately 80 percent fetuses in the experimental groups appeared small for their age compared to only 9 percent fetuses in the control group, which may have been due to the accumulation in embryos. These results indicated that a large amount of Ag in maternal tissues and a small amount in embryos may impact embryonic growth, but the mechanism is unclear [67].

Exposure to Ag NPs increases the risk of damage to the nervous system of fetuses. Thirty first-time pregnant NMRI mice were subcutaneously administered with 0, 0.2 and 2 mg/kg of body weight of Ag NPs once every three days from gestation day 3 until delivery. The Morris water maze (MWM) test showed that the spatial learning and memory of the offspring were impaired, which indicated that prenatal exposure to Ag NPs might have adverse effects on the central nervous system development in the fetus [68]. Intraperitoneal injection of Ag NPs (20-50 nm, 0.427 mg/g) every two days from GD10 to GD18 in prenatal rats also demonstrated that Ag exposure in maternal rats could result in cognitive impairment in the offspring. Histological assessment showed that the hippocampal structure of the offspring was deformed compared to those in control groups, and the MWM test demonstrated impaired spatial cognition in the male offspring [69]. Sprague Dawley female rats were orally administered with Ag NPs (7.9 ± 0.95 nm) at a dosage of 250 mg/kg from 14 days before mating to the fourth day after delivery (along with synchronous lactation). The results of ICP-MS and electron microscopy showed that the Ag NPs had abundantly accumulated in the tissues of the offspring compared with the tissue in the control groups (12.3-fold in the kidneys, 7.9-fold in the liver, 5.9-fold in the lungs, and 5.4-fold in the brain), which also indicated that the possible transfer of Ag NPs from pregnant rats to offspring occurs mainly through the placenta or breastmilk [27].

The size and coating of metal NPs are important factors in determining whether they can penetrate the PB and result in developmental toxicity. Three different-sized Au NPs (3 nm (Au3), 13 nm (Au13) and 32 nm (Au32)) were administered to pregnant mice with intrauterine inflammation. The results showed that Au3 and Au13 NPs could cross the PB and accumulate in the fetuses, resulting in increasing intrauterine inflammation.

However, Au32 NPs could not cross the PB in either the mice with intrauterine inflammation or healthy mice, which indicated that particle size is an important factor in the penetration of PB [70], consistent with the results of Yang et al. (2014). The surface composition of metal NPs also showed differences in different placental damage and fetotoxicity. Pregnant mice were intravenously administered with 13 nm Au NPs with the following three types of surface modifications: Au-Ft (coated with ferritin, treatment concentration 1.1*10^17^/ml), Au13-PEG (coated with PEG, treatment concentration 8.3*10^12^/ml) and Au13-CT (coated with citrate, treatment concentration 8.3*10^12^/ml) at E5.5-15.5. The amount of accumulated Au-Ft and Au13-PEG NPs was significantly greater than that of Au13-CT NPs in the fetus and extraembryonic tissues; however, none of the groups showed developmental toxicity in the fetuses [55].

### Nano oxide (TiO_2_)

Adult female rats gavaged with 100 mg/kg TiO_2_ NPs (10 nm) from GD2-21 exhibited effects on offspring in various aspects, such as memory impairment and decrease in hippocampal cell proliferation [71, 72], while oral treatment with the same dose during lactation on the same days resulted in a decline in spatial recognition memory and deficiency in learning of offspring [73]. Exposure to polyalcohol-coated TiO_2_ dust (21 nm) via inhalation for 1 h/day at a concentration of 42 mg/m^3^ could induce long-term lung inflammation accompanied by differential cell counts of bronchoalveolar lavage fluid in pregnant mice and cause moderate neurobehavioral alterations in the offspring, while the cognitive function was unaffected [57]. Prenatal TiO_2_ (∼25-75 nm) exposure, via injection of 100 µl/time four times on days 6, 9, 12 and 15, could result in alteration of the olfactory bulb, cerebral cortex and regions related to the dopamine systems in the offspring. Gene expression in the offspring was also dysregulated, including genes related to the striatum, dopamine neuron system and the prefrontal lobe [74]. Pregnant mice were exposed to 42 mg of titanium dioxide (UV-Titan)/m^3^ via inhalation for 1 h/day from GD8 to GD18. A comet assay revealed that the pregnant mice and offspring did not have any DNA strand breaks. While transcriptional profiling of offspring livers demonstrated that the expression of genes related to the retinoic acid signaling pathway was changed in the female offspring, which indicated that the inhaled UV-Titan could cross the PB and disturb gene expression of the offspring [75]. Coincidence with the abov study that slight neurobehavioral alterations were noted in the fetus of mice treated with UV-Ti NPs (of an average size of 21 nm) via inhalation [57]. The same study also found that TiO_2_ NPs could affect the development of the fetal central nervous system [58].

Silica particles nSP70, nSP300 and mSP1000 and TiO_2_ NPs of diameters of 70 nm, 300 nm, 1000 nm and 35 nm, respectively, were intravenously injected into the placenta at a dose of 0.8 mg per mouse. Transmission electron microscope (TEM) analysis revealed that the nSP70 NPs could accumulate in the fetus and maternal placenta, and the TiO_2_ NPs had also accumulated in the fetal liver and brain and placental trophoblasts. The mice treated with 70 nm silica particles and 35 nm TiO_2_ NPs showed 20% and 30% respective decrease in uterine weight, smaller fetuses (∼10%) and smaller amniotic sacs. A TdT-mediated dUTP Nick-End Labeling (TUNEL) assay indicated that nSP70 could induce apoptotic cell death of spongiotrophoblasts resulting in variable structural abnormalities of the mouse placenta. However, the silica particles in the other groups exhibited no accumulation in the placenta, fetal liver or fetal brain and displayed no fetotoxicity or placental dysfunction. All of these results indicated that nSP70 and 35 nm TiO_2_ NPs might affect maternal-fetal exchange, resulting in fetal resorption and restricted fetal growth. Therefore, these detrimental effects of NPs in fetuses and placenta may be linked to the size of NPs [76].

### Nano oxide (Iron oxide)

With the increase in the application of iron oxide NPs in photocatalysis, drug delivery and biomedical imaging, the risk of exposure to iron oxide NPs in humans, especially pregnant women and developmental fetuses, is of great concern. To assess the reproductive toxicity of Fe_3_O_4_ NPs, dimercaptosuccinic acid (DMSA)-coated Fe_3_O_4_ NPs were intraperitoneally injected into pregnant mice. At doses higher than 50 mg/kg, the weight of infants showed a significant decrease, and the male pups exhibited significant decrease in spermatogenic cells (spermatogonia, spermatocytes, spermatids and mature sperm) by testicular histological results, which indicated that DMSA-coated Fe_3_O_4_ NPs can disrupt embryonic development at doses higher than 50 mg/kg [77].

CD-1 mice were intraperitoneally injected with PEI-Fe_2_O_3_ NPs (∼28 nm, zeta potential: 51 mV) or PAA-Fe_2_O_3_ NPs (∼30 nm, zeta potential: -52 mV), which were coated with the hydrophilic ligands polyethyleneimine (PEI) or poly(acrylic acid) (PAA), respectively. The results indicated that both positively charged PEI-Fe_2_O_3_ NPs and negatively charged PAA-Fe_2_O_3_ NPs had the ability to cross the placenta and accumulate in the fetus, though multiple doses of positively charged PEI-Fe_2_O_3_ NPs resulted in significantly accumulation of iron in the placenta and increased fetal death over several days [78].

Whether Fe_2_O_3_ NPs can cross the human PB also depends on their diameter. *In vitro* analysis showed that large (50 and 78 nm) but not small (15 nm) α-Fe_2_O_3_ could increase cell death and ROS resulting in disruption of BeWo epithelial barrier functions [79].

### Nano oxide (Zinc oxide)

To study the effects of ZnO NPs, pregnant rats were treated with 500 mg/kg body weight of ZnO NPs of less than 100 nm in diameter. The ZnO NPs were distributed in mammary tissues of the dams together with the liver and kidneys of pups. The accumulation of ZnO NPs could increase fetal resorption and decrease the body weight of pups [80]. Liu *et al.* found that ZnO NPs exposure (ZnSO_4_-200 mg/kg and ZnO-NP-200 mg/kg of diet) in hens could increase γ-H2AX and decrease NF-κB to decrease cell proliferation or increase apoptosis in embryos. The underlying reason was that ZnO NPs may damage DNA replication and repairmen machinery in hen oocytes, which subsequently inhibited embryonic development [81].

### Nano oxide (Cadmium oxide)

To detect the toxicity of cadmium oxide (CdO) NPs in pregnant mice and offspring, pregnant CD-1 mice were exposed to CdO NPs (15.3 nm ± 1.6 nm, 230 μg/m^3^) via inhalation from GD4.5 through GD16.5, and the nephrotoxic indexes were examined. The results showed that mRNA expression of kidney injury molecule-1 (Kim-1) in the urinary tract was five-times higher in the pregnant mice at GD10.5 and 3.2-times higher in the neonatal offspring at PND14 than the levels in the corresponding controls, which demonstrated that inhaled CdO NPs could enter circulation and penetrate the PB resulting in their accumulation and renal injury in fetuses and mothers [82]. Similarly, a study on CdO NPs by inhalation also demonstrated that the decrease in fetal weight was due to the accumulation of NPs in the placenta [83].

### Nano oxide (Cupric oxide)

To simulate the *in vivo* human placenta, a well-organized 3D co-culture microtissue (MT) model was developed, which consisted of placental fibroblasts surrounded by a trophoblast cell layer. The results showed that incubation with CuO NPs (4 nm) could decrease the viability of the MT and reduce the release of human chorionic gonadotropin (hCG) [84]. Additionally, the *ex vivo* human placental model provides a convenient method for studying the mechanism by which NPs cross the human PB.

### Carbon nanomaterials

Exposure of prenatal mice to carbon black NPs (CB-NPs; 95 mg/kg; particle size: 14 nm; surface area: 300 m^2^/g) by intranasal instillation on GD9 and GD15 could increase total thymocyte and total lymphocyte counts, promote immune responses and alter gene expression associated with the induction of peripheral tolerance in male offspring. These results indicated that respiratory exposure to CB-NPs during pregnancy may result in inflammatory or allergic effects in the offspring [85]. Moreover, intranasal instillation of CB-NPs in pregnant mice on GD5 and GD9 could decrease CD3+ (T), CD4+ and CD8+ T cells in the spleens of 1- to 5-day-old offspring. In addition, CB-NPs could also significantly upregulate the expression level of ll15 in the newborn male offspring as well as Ccr7 and Ccl19 in the female offspring. These results indicated that exposure of pregnant mice to CB-NPs may suppress the development of the immune system in the offspring [86].

Exposure of pregnant mice to CB-NPs (95 mg/kg; particle size: 14 nm) by intratracheal administration on GD7 and GD14 resulted in partial vacuolation of seminiferous tubules and reduction in cellular adhesion of seminiferous epithelia and in daily sperm production (DSP) in male offspring. In addition, the body, testis and epididymis weights and concentration of serum testosterone were also different between the control and CB-NP groups, which suggested that exposure to CB-NPs could affect the reproductive function of male offspring [87]. In another study, 200 μg of 14-nm carbon NPs was administered by inhalation into pregnant mice on days 7 and 14. The daily sperm production was significantly decreased in the carbon nanoparticle-exposed fetuses [87].

### Quantum Dots (QDs)

Currently, quantum dots are gaining popularity in their application in both biomedical imaging and targeted therapeutics. Whether QDs are transferred from the pregnant mother to its fetus may depend on their size, dose, charge, capping material properties and inorganic components.

To evaluate the clinical safety of CdTe/CdS QDs, Chu et al. studied pregnant mice that were intravenously injected with four different doses (20, 50, 86, or 125 mg), three different sizes (MPA-coated QDs (532), MPA-coated QDs (599), MPA-coated QDs (656)), and three different coatings (MPA-coated, PEG-coated and SiO_2_-coated QDs (656)) of Cd-containing QDs. The results showed that abundant Cd atoms derived from the QDs were accumulated in the pups, and the amount of Cd atoms increased with increasing doses of QDs. An analysis of the size dependence of Cd-QD NPs demonstrated that smaller QDs were more efficiently transferred into the pups, which implied the presence of extracellular transport pathways across the placenta. Assessment of three different coatings of Cd-QDs showed that the average concentration of Cd in the MPA-coated QD-treated pups was higher than that of the PEG-coated and SiO_2_-coated QD-treated groups. The authors of this study concluded that the PB could not prevent Cd-QDs from entering the pups, and the ability of QDs to travel from the mothers to pups depend on the dosage, size and coating components [88]. Mice were intraperitoneally injected with PEG-coated CdSe QDs (4 nm) suspended in PBS at the dosage of 150 μg/kg body weight (containing 3.75 μg Cd/Te-cores) or 750 μg/kg body weight (containing 18.75 μg Cd/T-cores) every 3 days for 5 weeks. A small amount of Cd was found in the placenta of the high concentration group, which indicated that the ability of the PEG-coated CdSe QDs to cross the PB was dose-dependent [89].

Pregnant mice were injected with CdSe/ZnS QDs (∼20 nm) or CdSe QDs (∼15 nm) at a concentration of 0.5 µM, 100 µL/mice/day at GD16 and GD17. RT-PCR assay showed that in both treatment groups, the expression of symbolic genes, such as p53 and bax (related to apoptosis), IGF-1 and EGF (related to tissue development), MT-1 (related to metal transport), was significantly altered. The CdSe/ZnS QD-treated group exhibited milder toxicity compared with the CdSe QD-treated group, which could be attributed to the protection of the ZnS surface coating [90]. The results from another previous *in vitro* study were consistent with the finding above. To assess the cytotoxic effect of CdSe-core QDs on embryonic development, blastocysts were obtained from mouse uterine horn on the fourth day of pregnancy and treated with CdSe-core QDs (0, 25, 250 and 500 nmol/L) for 24 h. TUNEL staining analysis showed that mouse blastocysts underwent apoptosis in response to CdSe-core QD in a dose-dependent manner. Dual differential staining revealed that proliferation in blastocysts was decreased by CdSe-core QDs, resulting in the inhibition of post-implantation embryonic development. However, the ZnS coating of CdSe QDs could significantly decrease these cytotoxic effects on embryonic development [91].

Generally, the PB is formed at approximately GD10-12 in mice and at approximately 4 months of pregnancy in humans [27]. After the formation of PB, the toxic NPs with less than 50nm particles showed injury to placenta and accumulated in the fetus, which indicating that less than 50nm of NPs can penetrate the PB. While, the size of NPs penetrated the BTB is smaller than 4 nm. Compared with BTB, the penetrability of PB may be attribute to its function, which is an important system in the exchange of nutriment or excretory metabolism between the mother and fetus.

**Figure 3:**
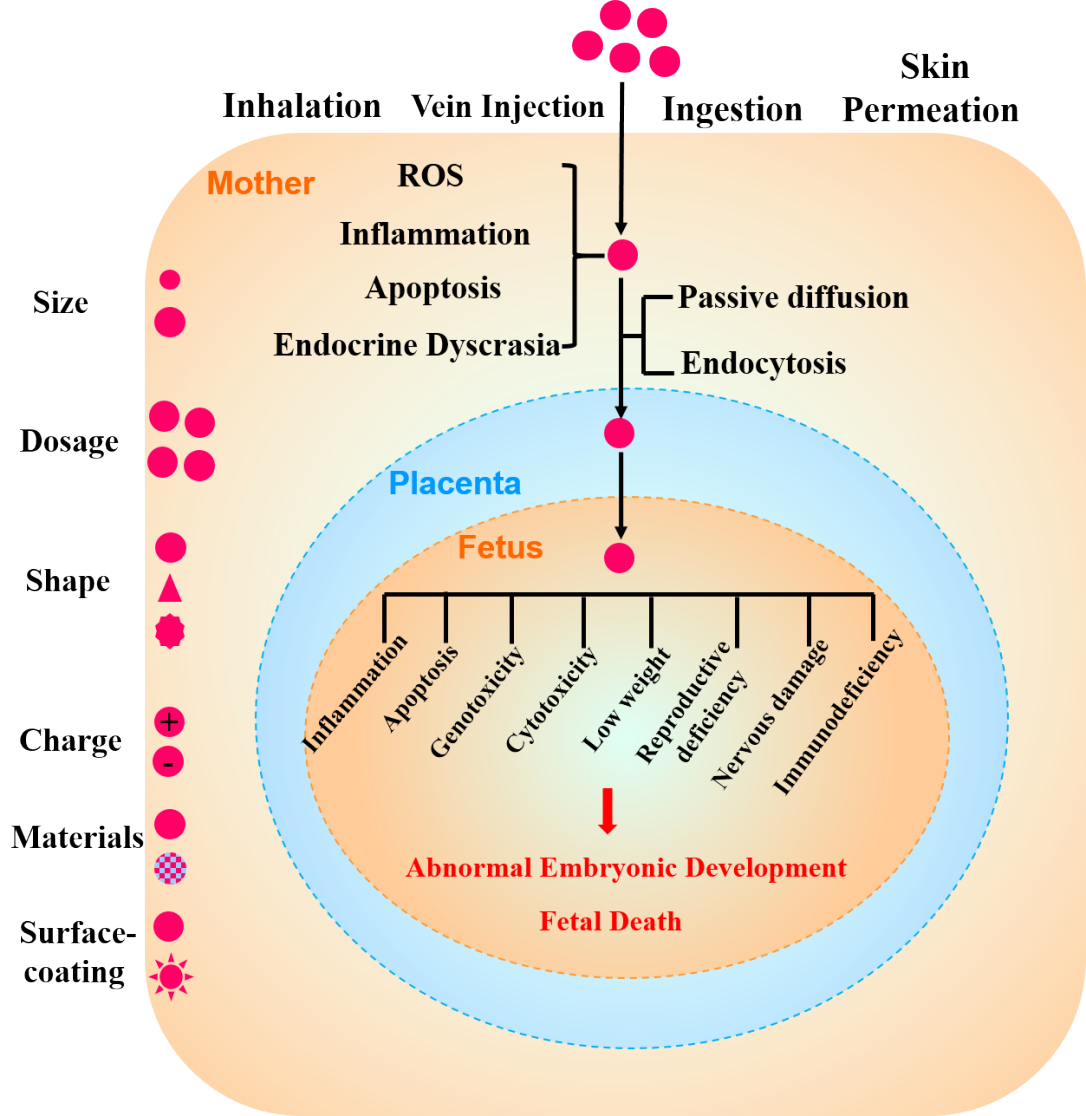
The toxicity of NPs in pregnancy and embryonic development Factors affecting the toxicity of NPs are their size, dosage, shape, charge, material and surface-coating. After NPs enter a pregnant woman’s body through inhalation, venous injection, ingestion or skin permeation, maternal toxic stress reactions such as ROS, inflammation, apoptosis and endocrine dyscrasia are induced. During pregnancy, NPs may across the placenta into the fetus by passive diffusion or endocytosis. The toxic effect of NPs can trigger fetal inflammation, apoptosis, genotoxicity, cytotoxicity, low weight, reproductive deficiency, nervous damage, and immunodeficiency, among other mechanisms, which can result in abnormal embryonic development and fetal death.

In summary, damage by the NPs to the PB and embryo is related to the following aspects: 1. the physicochemical properties of NPs (size, dosage, shape, charge, material and surface-coating). There is a negative correlation between the rate of passage through the PB and size of the nanomaterials, which may be closely related to the pore size of the placental barrier. Surface modification materials affect the penetration of NPs by changing their physicochemical properties. After NPs enter a pregnant woman’s body through inhalation, venous injection, ingestion or skin permeation, maternal toxic stress reactions such as ROS, inflammation, apoptosis and endocrine dyscrasia are induced. During pregnancy, NPs may across the placenta into the fetus by passive diffusion or endocytosis. The toxic effect of NPs can trigger fetal inflammation, apoptosis, genotoxicity, cytotoxicity, low weight, reproductive deficiency, nervous damage, and immunodeficiency, among other mechanisms, which can result in abnormal embryonic development and fetal death (Figure 3). 2. the health of the uterus, NPs are more likely to pass through the PB in an inflamed uterus. Thus, more attention should be paid to avoid exposure to NPs, especially in pregnant females. These toxicity profiles of NPs in embryonic development will provide the latest information for reasonable applications of NPs.

## THE POSSIBLE WAYS FOR NPS ACROSS THE PB

### Passive diffusion

By TEM analysis, Kertschanska et al. (1997) demonstrated that the placental channels that begin from the basal trophoblastic plasmalemma and terminate on the maternal surface range from 15-25 nm in diameter under normal intravascular pressure [92]. Therefore, this pore size would allow the NPs of size under 25 nm to cross the PB by passive diffusion.

It was demonstrated that NPs of polystyrene beads with 50, 80 or 240 nm in diameter could cross the barrier without impairing its function. In contrast, 500-nm beads were significantly blocked. The authors suggested that the fast transport of NPs with diameters of 50 and 80 nm may occur by diffusion [64]. These results are similar to those of previous studies on rodent placenta where size was also a factor. Silicon NPs with a size of 519 nm passed the PB, while those of sizes 834 and 1000 nm were unable to penetrate it [93]. The permeability of NPs is also related to the various NP coatings. Myllynen et al. studied PEGylated gold NPs in both open and recirculating dual human placental perfusion systems. These NPs aggregated in the syncytiotrophoblast cell layer rather than crossing the PB [60].

### Endocytosis

A recent study showed that the intravenous administration gold NPs (20-nm and 50-nm NPs) were transported by endocytosis into placental cells [62]. Chun et al. also showed that QDs could be internalized by endocytosis and then migrate into fetal blood through fetal capillary pores [88]. The possible mechanism of endocytosis was related to clathrin and caveolin as the placenta has increased expression of clathrin and decreased expression of caveolin [62]. Therefore, although the mechanism of NP transport through the placenta is not fully understood, according to existing results, the main pathway of NP transport seems to be that of endocytosis [62]. In addition, diffusion has also been mentioned to be related to size-dependent transport of NPs. Selective permeability and passive and active transport must also be taken into account. Another possible mechanism relating to cell structure, called the ‘transtrophoblastic channel’, has also been suggested [64]. Due to the different results in various type, coating, or diameter of NPs, several of the above mechanisms may need to be considered in combination. The toxicity of NPs in pregnancy and embryonic development is shown in Figure 3 [63, 64]. The mechanisms that are responsible for the transport of NPs across the PB should be investigated to prevent damage to the development embryos. Meanwhile, understanding of the molecular transport mechanisms is important for accelerating the development of new therapeutic tools.

However, there is some evidence suggesting that certain NPs cannot pass through the PB. CdTe QDs coated with polyethylene glycol or mercaptopropionic acid, for example, were injected into Wistar rats, and subsequent penetration through the placental barrier was observed [94]. These QDs were mostly found in the complex cell structure of the placenta [94]. PEGylated gold NPs (size: 10-30 nm) could not cross the perfused human placenta in detectable amounts into the fetal circulation within 6 h [60]. In future studies of transport of NPs across the PB, rodent placenta and placenta perfusion models may be efficient techniques to investigate the transport of NPs between the mother and fetus [61, 63, 83].

Unfortunately, recent data on the cellular mechanisms underlying the variable permeability of NPs across the PB are still unclear. Thus, there is a need to balance the application of the NPs during pregnancy to prevent impeding embryogenesis.

## CONCLUSIONS AND FUTURE PERSPECTIVES

With the increased application of engineered NPs in cosmetics and textiles, together with the inhalation of NPs polluting the air, understanding the reproductive toxicity and transferable adverse effects of next generation is necessary. Interestingly, NPs are a double-edged sword: on one hand, owing to their peculiar physical and chemical properties, NPs can be used in commodities (such as cosmetics and textiles), in drug delivery systems and in clinical therapy and can provide substantial advantage and effectiveness in these fields [95]; on the other hand, due to the non-degradable properties of NPs, increasing evidence from research has shown the potential of many adverse health effects relating to the use of or contact with NPs. Many of these concerns have been highlighted from both *in vivo* and *in vitro* studies. However, while the studies on the toxicity of NPs have been far fewer than those on the positive application of NPs, the small size of NPs allows them to easily enter the body, particularly via the skin, eyes, gastrointestinal tract and nasal olfactory structures, at which point many potential harmful effects may occur. Subsequently, these NPs can enter systemic circulation and traverse tissues, cells and organelles as well as the reproductive organs [96, 97].

This review has discussed the present knowledge on the influence of NPs on oogenesis, ovarian structure, hormonogenesis and embryonic development (Table 1-4). Recent studies have shown that NPs can cause genotoxicity, cytotoxicity and abnormal development of the embryo. With the application of ENPs in commercial and industrial products, the risk of environmental exposure is growing. However, it is rather difficult to compare reproductive toxicity between studies, especially because the variety and dosage of NPs are very diverse. The application of NPs in pregnant women and fetuses still needs more in-depth studies. This review also provided a reference for the application of NPs in the clinic for gynecological disease.

## Abbreviations

NPs: nanoparticles
ROS: reactive oxygen species
BBB: blood-brain barrier
TiO2: titanium dioxide
CNS: central nervous system
GnRH: gonadotropin-releasing hormone
FSH: follicle stimulating hormone
LH: luteinizing hormone (272)
QDs: quantum dots
QD-Tf: quantum dot-transferrin
IVM: *in vitro* maturation
ZP: zona pellucida
IVF: *in vitro* fertilization
ENPs: engineered nanoparticles
BMF: BCL2 modifying factor
SOD: superoxide dismutase
CAT: catalase
MDA: malondialdehyde
HPOA: hypothalamic-pituitary-ovarian axis
PEG-b-PLA: poly(ethylene glycol)-block-poly(lactic acid)
P4: progesterone
E2: estradiol
SCC: side-chain cleavage
HA: hydroxyapatite
GPx: glutathione peroxidase
GD: gestation day
VYS: visceral yolk sac
MWM: Morris water maze
PB: placental barrier
TEM: transmission electron microscope
TUNEL: TdT-mediated dUTP Nick-End Labeling
DMSA: dimercaptosuccinic acid
PEI: polyethyleneimine
PAA: poly(acrylic acid)
CdO: cadmium oxide
Kim-1: kidney injury molecule-1
PND: postnatal days
MT: microtissue
hCG: human chorionic gonadotropin
CB-NPs: carbon black NPs
DSP: daily sperm production
